# Aggressive Malignant Paraganglioma Involving the Pancreas and Vertebral Column

**DOI:** 10.7759/cureus.40985

**Published:** 2023-06-26

**Authors:** Salahudin Mahmood, Abhilasha N Borkar, Farhan A Khan, Tammey Naab

**Affiliations:** 1 Internal Medicine, Nishtar Medical University and Hospital, Multan, PAK; 2 Pathology and Laboratory Medicine, University of Tennessee Health Science Center, Memphis, USA; 3 Pathology and Laboratory Medicine, Pathology Specialists of Memphis, Memphis, USA; 4 Pathology and Laboratory Medicine, Methodist Le Bonheur Healthcare, Memphis, USA; 5 Pathology and Medical Microbiology, Athari Bio + Sciences, Washington DC, USA

**Keywords:** germline mutations, hereditary, sdh deficient, paraganglioma, aggressive

## Abstract

Paraganglioma (PGL) is a rare neuroendocrine tumor arising from chromaffin cells outside the adrenal medulla. The most common sites are the abdomen and head and neck. Seventy percent (70%) of PGLs are sporadic, and 30% are hereditary; the latter are more often aggressive and malignant and occur in young adults. We report a case of a 36-year-old woman with a history of hypertension and abdominal pheochromocytoma resected at the age of 10 years who presented with back pain. Magnetic resonance imaging of the spine showed vertebral metastasis at L2-L5. Computed tomography of the abdomen showed a mass in the body of the pancreas and a laparoscopic biopsy was performed. The tumor cells had granular eosinophilic/basophilic cytoplasm and showed a nested pattern (Zellballen) with a prominent vascular network and infiltration of dense fibrous connective tissue. Strong and diffuse expression of synaptophysin in tumor cells, S100 expression in sustentacular cells at the periphery of nests, and lack of pancytokeratin expression supported the diagnosis of PGL. Due to limited tissue, it was difficult to determine metastatic vs primary neoplasm of the pancreas. The earlier age of onset and history of abdominal pheochromocytoma suggested the possibility of hereditary PGL associated with succinate dehydrogenase (SDH) deficiency. The tumor cells lacked SDHB expression. Germline mutation testing for SDH was recommended. The patient underwent palliative radiotherapy and systemic chemotherapy. Most PGLs are benign and asymptomatic, but there is an increased risk of cardiovascular mortality secondary to catecholamine secretion, and surgical excision is curative. Malignant PGLs are rare (10-40%), have poor prognosis, and are incurable. Increased size of the tumor, deep tissue infiltration, and high proliferative index increase the risk of malignancy, but metastasis is required for the diagnosis of malignant PGL. The advanced disease is treated with surgical removal of the tumor and combined radiotherapy and chemotherapy.

## Introduction

The World Health Organization (WHO) classifies pheochromocytoma and paraganglioma as rare neuroendocrine tumors that arise from chromaffin cells of the adrenal medulla and extra-adrenal sympathetic and/or parasympathetic paraganglia, respectively [1,2].

Paraganglioma (PGL) is a rare neuroendocrine tumor arising from chromaffin cells in sympathetic and parasympathetic ganglia outside the adrenal medulla. The most common sites are the abdomen followed by the head and neck. Seventy percent (70%) of paragangliomas are sporadic and 30% are hereditary; the latter are more often aggressive and malignant and occur in young adults [3].

Catecholamine-secreting paraganglioma may present with symptoms of catecholamine excess while those that are nonfunctioning may lead to local compressive symptoms. Paragangliomas account for about 0.3% of all neoplasms. The majority of paragangliomas are benign tumors while almost 15-20% are metastatic. Metastases are present at the initial diagnosis in about 11-31% of patients. Five-year survival rates range from 40-85% [4].

The diagnosis of metastasis is based on the presence of tumors in sites that lack the presence of chromaffin cells. Hereditary PGLs are associated with germline mutations in the succinate dehydrogenase (SDH) gene [5,6].

This article was previously presented as a meeting abstract at the 2016 ASCP Annual Scientific Meeting on September 14, 2016

## Case presentation

**Figure 1:**
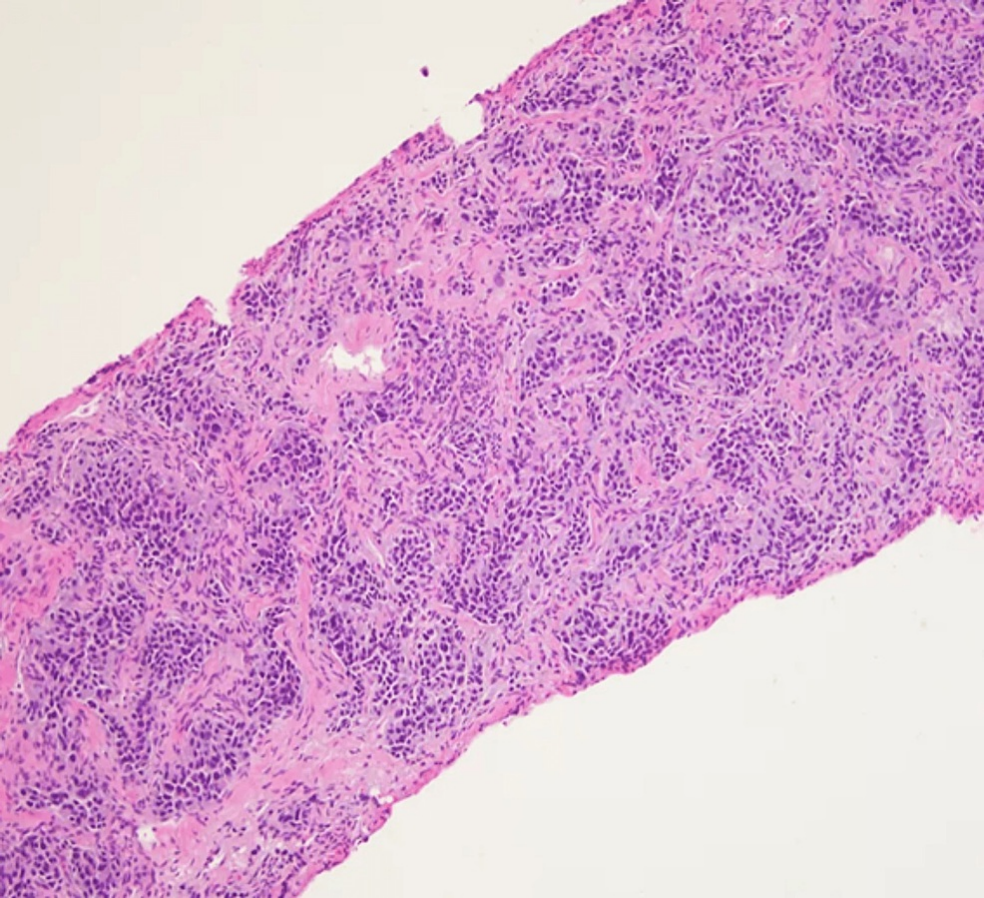
Tumor cells infiltrating the stroma in a nested pattern (x100)

**Figure 2:**
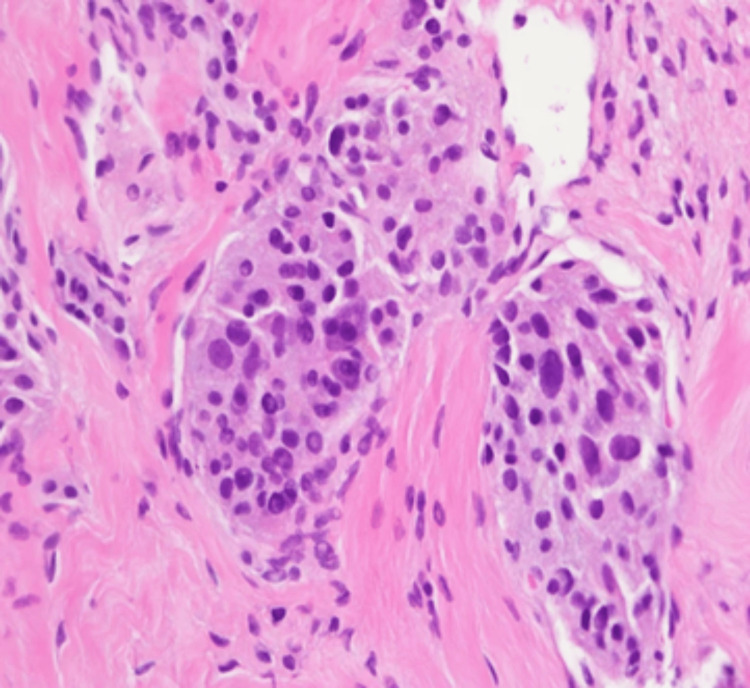
Nests of tumor cells with eosinophilic to basophilic granular cytoplasm and nuclear atypia (x400)

**Figure 3:**
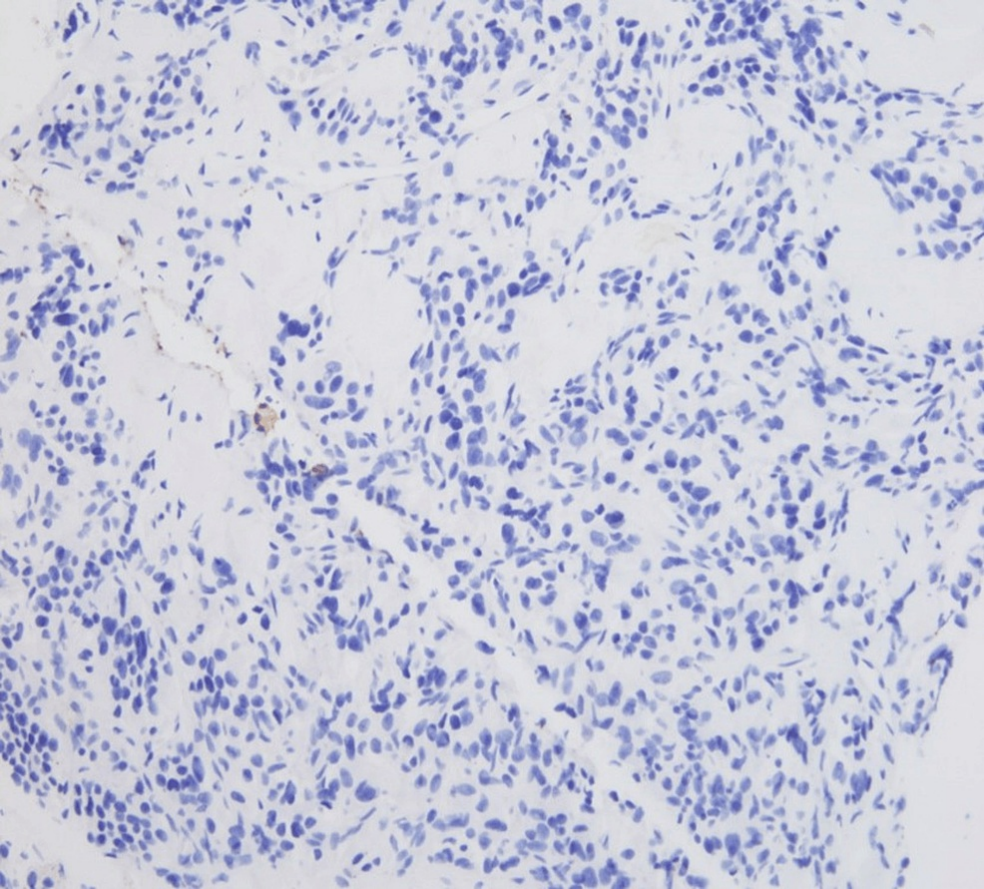
Tumor cells show a lack of pancytokeratin expression (x200)

**Figure 4:**
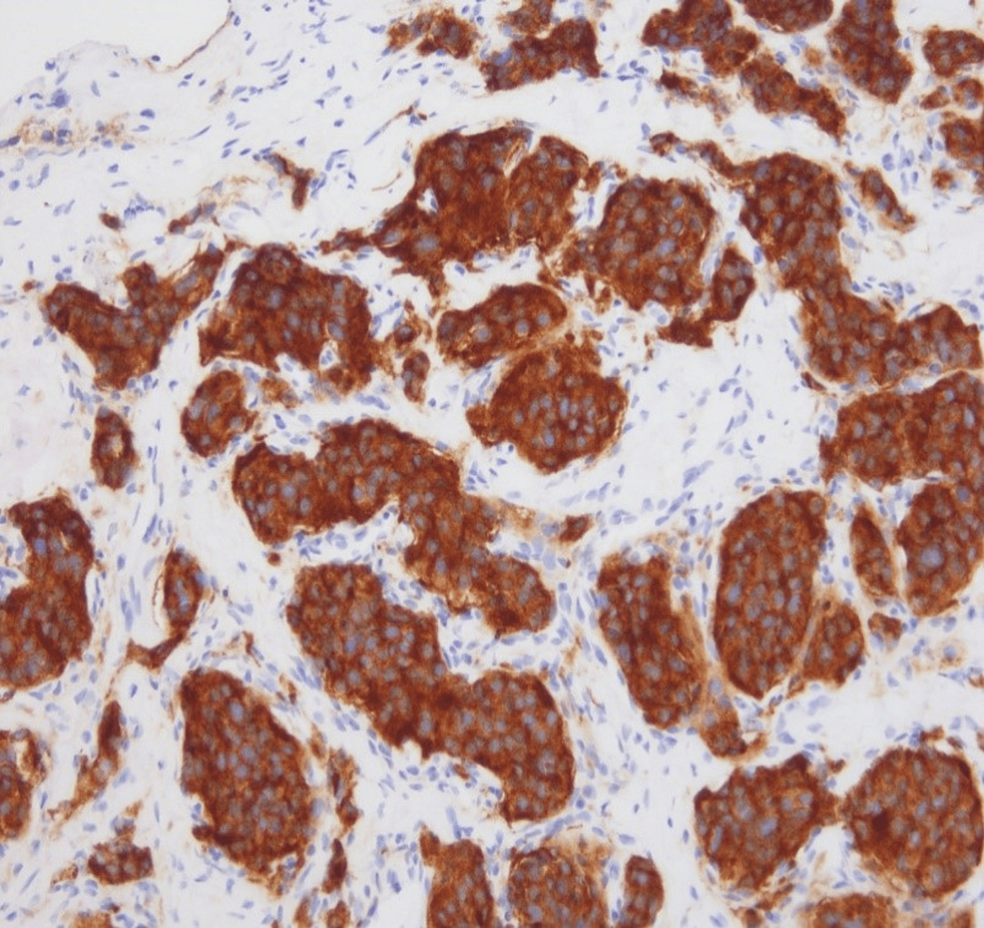
Tumor cells show strong and diffuse expression of synaptophysin (x200)

**Figure 5:**
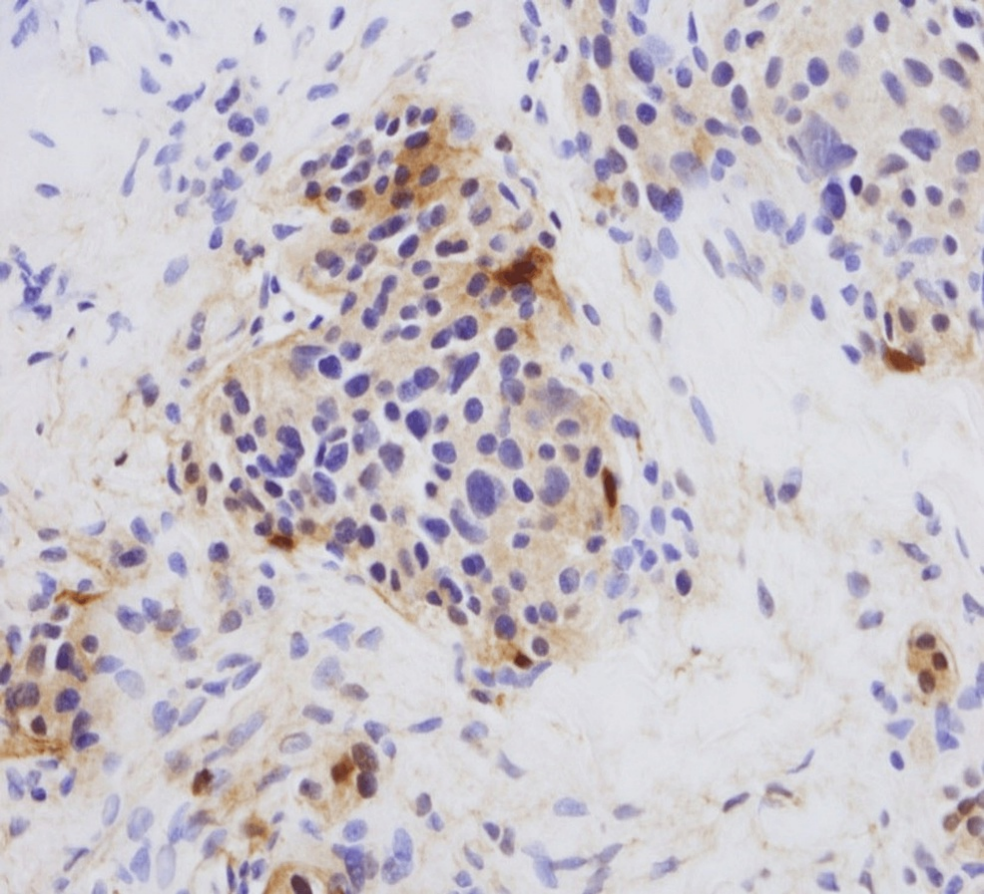
S100 stain of sustentacular cells (x200)

We report a case of a 36-year-old female with a history of abdominal pheochromocytoma status post resection at the age of 10 years, who presented with back pain. MRI of the spine was concerning for metastases involving L2-L5. CT of the abdomen showed a mass in the body of the pancreas with subsequent laparoscopic biopsy. The tumor cells showed the characteristic histology of paraganglioma with a nested pattern (Zellballen), accompanying prominent vascular network, and dense granular cytoplasm (Figures 1, 2). Tumor cells were nonreactive to cytokeratin and showed diffuse reactivity with synaptophysin (Figures 3, 4). S100 showed expression in sustentacular cells (Figure 5). Immunophenotypic findings were consistent with paraganglioma (PGL). Due to limited tissue, it was difficult to determine metastatic versus primary neoplasm of the pancreas. No normal pancreatic tissue was seen with PGL.

**Figure 6:**
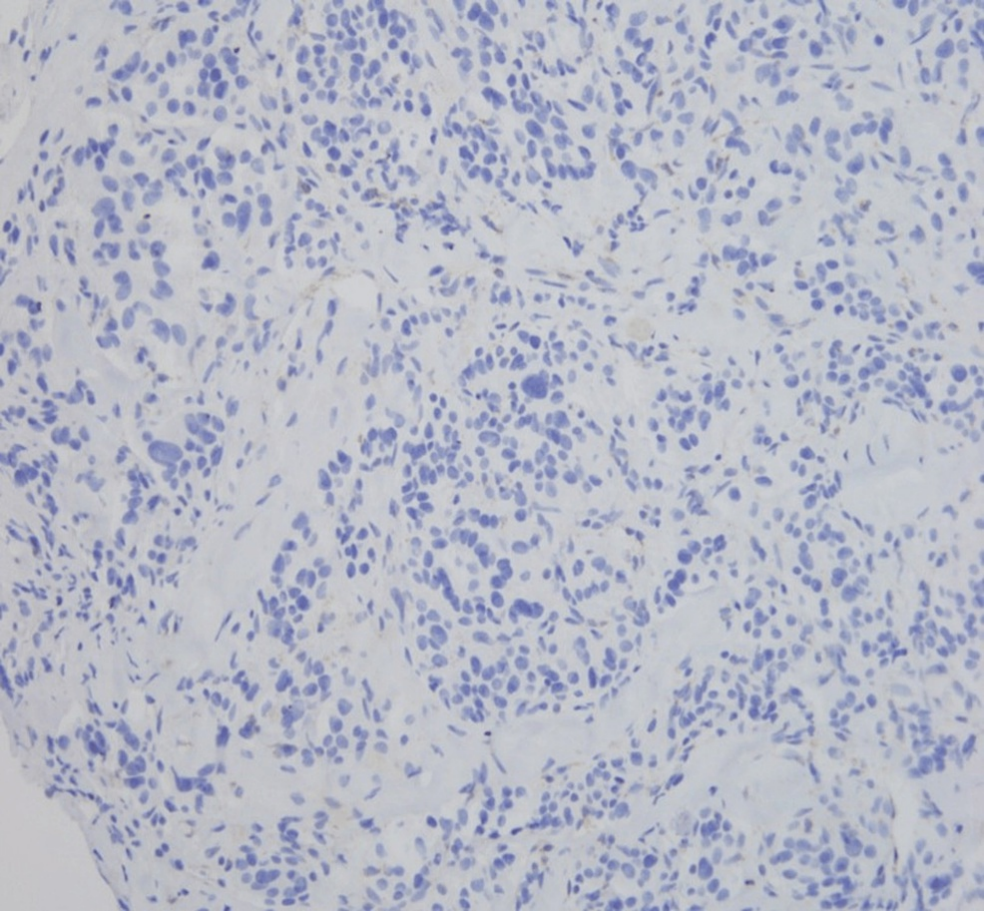
Tumor cells negative for SDHB (x200)

The possibility of hereditary PGL associated with succinate dehydrogenase (SDH) deficiency was considered and tumor cells lacked SDHB expression (Figure 6). Germline mutation testing for SDH was recommended. The patient underwent palliative radiotherapy and systemic chemotherapy.

## Discussion

Paragangliomas are clusters of neuroendocrine cells derived from neural crests. They are associated with the sympathetic as well as the parasympathetic nervous system [7]. They might undergo neoplastic transformation into a paraganglioma. Paragangliomas are most commonly seen within the adrenal medulla, giving rise to pheochromocytoma. Outside the adrenal gland, they are usually seen in paravertebral paraganglia and paraganglia near the great vessels of the head and neck [8].

Bony metastases in paragangliomas can occur in the ribs, vertebrae, or pelvis. A few cases of mandibular metastases have also been reported [9].

Elevated plasma and urine metanephrine levels are useful for diagnosing PGL. Most PGLs are benign and asymptomatic, and surgical excision is curative. The SDHB-deficient PGL associated with hereditary PGL is more prone to metastasis [6]. The advanced disease has limited therapeutic options. Hereditary PGL has an increased risk of gastrointestinal stromal tumors, renal clear cell carcinoma, and papillary thyroid carcinoma.

There is no definitive curative therapy for patients having metastatic disease. The goals of treatment include reducing tumor size, controlling the catecholamine excess symptoms with alpha receptor blockers, catecholamine receptor inhibitors, and palliative symptoms because of the tumor burden.

Paragangliomas involving the spine are quite rare. Most of them are primary tumors arising from the spine while only a few are those that metastasized to the spine [10].

Immunostains for synaptophysin, chromogranin A, or S100 protein can be targeted for the identification and confirmation of paraganglioma. Genetic studies show that mutations in SDHB, SDHC, and SDHD are associated with transmissible germline mutations in which future generations have a higher risk for pheochromocytoma and paraganglioma. These genes are tumor suppressor genes that encode for subunits of mitochondrial complex II; mutations lead to pheochromocytoma paraganglioma syndrome [11].

The mainstay treatment for paraganglioma is surgery and is especially important for those who have metastasized to the spine. as these tumors cause significant mass-effect leading to neurological deficits and pain.

There is a lack of studies that examined the role of chemotherapy and radiotherapy for spinal paragangliomas. Some studies suggest the role of somatostatin analogs and octreotide as an effective complementary treatment for paragangliomas. If surgery is not an option, radiotherapy and systemic anticancer octreotide can be effective for paragangliomas of the spine [12]. A study has been presented in which a 32-year-old female patient was treated without surgery using a combination of octreotide and radiotherapy [13].

## Conclusions

This case study highlights the importance of performing SDHB testing in patients with PGL of early onset and unusual site involvement for excluding hereditary PGL. Germline mutation testing in SDHB, SDHC, and SDHD can be performed, which is associated with transmissible germline mutations in which future generations have a higher risk for pheochromocytoma and paraganglioma. Further clinical studies are warranted to clarify the roles of molecularly targeted therapy.

## References

[1] Fliedner SM, Lehnert H, Pacak K (2010). Metastatic paraganglioma. Semin Oncol.

[2] Lam AK (2017). Update on adrenal tumours in 2017 World Health Organization (WHO) of endocrine tumours. Endocr Pathol.

[3] O'Riordain DS, Young WF Jr, Grant CS, Carney JA, van Heerden JA (1996). Clinical spectrum and outcome of functional extraadrenal paraganglioma. World J Surg.

[4] Corssmit EP, Snel M, Kapiteijn E (2020). Malignant pheochromocytoma and paraganglioma: management options. Curr Opin Oncol.

[5] Hao HX, Khalimonchuk O, Schraders M (2009). SDH5, a gene required for flavination of succinate dehydrogenase, is mutated in paraganglioma. Science.

[6] Lodish MB, Adams KT, Huynh TT (2010). Succinate dehydrogenase gene mutations are strongly associated with paraganglioma of the organ of Zuckerkandl. Endocr Relat Cancer.

[7] Lingen MW (2009). Head and neck. Robbins & Cotran Pathologic Basis of Disease.

[8] Capella C, Riva C, Cornaggia M, Chiaravalli AM, Frigerio B, Solcia E (1988). Histopathology, cytology and cytochemistry of pheochromocytomas and paragangliomas including chemodectomas. Pathol Res Pract.

[9] Stanek J, Vahidi S, Wilke CT, Khaja SF (2019). Malignant paraganglioma presenting as a mandibular metastasis. Head Neck.

[10] Simpson LN, Hughes BD, Karikari IO, Mehta AI, Hodges TR, Cummings TJ, Bagley CA (2012). Catecholamine-secreting paraganglioma of the thoracic spinal column: report of an unusual case and review of the literature. Neurosurgery.

[11] van Nederveen FH, Gaal J, Favier J (2009). An immunohistochemical procedure to detect patients with paraganglioma and phaeochromocytoma with germline SDHB, SDHC, or SDHD gene mutations: a retrospective and prospective analysis. Lancet Oncol.

[12] Lau D, La Marca F, Camelo-Piragua S, Park P (2013). Metastatic paraganglioma of the spine: case report and review of the literature. Clin Neurol Neurosurg.

[13] U-King-Im JM, Carroll TA, Morris K (2002). Vertebral metastatic chemodectoma: imaging and therapeutic octreotide. Case report. J Neurosurg.

